# A Multimodal TinyML-Based Predictive Maintenance Architecture for Industrial IoT in the 6G Era

**DOI:** 10.3390/s26144536

**Published:** 2026-07-17

**Authors:** Carlos Exequiel Garay, Fernando Alberto Miranda Bonomi, Gonzalo Nicolás Mansilla, Mariano Fagre, Sergio Gustavo Guzmán, Pablo Alberto Ritorto, Franco Ismael Perez, Marcos Katz

**Affiliations:** 1 CIASUR (Centro de Investigación de Atmósfera Superior y Radiopropagación), Facultad Regional Tucumán (FRT), Universidad Tecnológica Nacional (UTN), Rivadavia 1050, San Miguel de Tucumán 4000, Argentina; exequielgaray@doc.frt.utn.edu.ar (C.E.G.); mansillagn@gmail.com (G.N.M.); 2 Laboratorio de Telecomunicaciones (LTC), Departamento de Electricidad, Electrónica y Computación, Facultad de Ciencias Exactas y Tecnología (FACET), Universidad Nacional de Tucumán (UNT), San Miguel de Tucumán 4000, Argentina; fmirandabonomi@herrera.unt.edu.ar (F.A.M.B.); mfagre@herrera.unt.edu.ar (M.F.); 3 Consejo Nacional de Investigaciones Científicas y Técnicas (CONICET), San Miguel de Tucumán 4000, Argentina; francoismael.perez@alu.frt.utn.edu.ar; 4 Laboratorio Área IV—Termología, Departamento de Mecánica, Facultad Regional Tucumán (FRT), Universidad Tecnológica Nacional (UTN), Rivadavia 1050, San Miguel de Tucumán 4000, Argentina; sergioguzman@doc.frt.utn.edu.ar (S.G.G.); pabloritorto@doc.frt.utn.edu.ar (P.A.R.); 5 Centre for Wireless Communications, University of Oulu, 90014 Oulu, Finland

**Keywords:** TinyML, predictive maintenance, anomaly detection, multimodal sensing, thermography, 6G, URLLC, Industry 5.0, sensor fusion, experimental validation

## Abstract

Predictive maintenance (PdM) is central to Industry 5.0 strategies for reducing unplanned downtime in rotating machinery. This work proposes and evaluates, as a proof of concept on a controlled single-machine testbed, a multimodal TinyML edge architecture for PdM designed to remain compatible across the application plane’s evolution toward sixth-generation (6G) networks. Three complementary modalities run local inference on commercial off-the-shelf smart sensor nodes—vibration, acoustic, and thermography—with an embedded gateway bridging per-modality decisions to a serverless cloud back-end. Using real vibration data from a controlled static-unbalance protocol, five anomaly-detection model variants, operating on ten frequency-independent time-domain features extracted from 6 s windows, are benchmarked on the actual Cortex-M4F target; the INT8-quantized fully connected autoencoder, scored by per-window reconstruction error, reaches F1 = 0.9807 with 254 µs inference latency and a 6056 B Flash footprint, well within the microcontroller budget. In a second acquisition session with the remounted sensor, the frozen model retains perfect fault recall, and a short per-installation healthy-baseline recalibration restores F1 = 0.975 without any weight retraining. The acoustic modality is classified in-sensor on log-Mel filterbank energies by the Syntiant NDP120 neural coprocessor, and the thermographic modality by a lightweight binary CNN on 96 × 96 px frames. A preliminary intra-session late-fusion analysis suggests that a logistic-regression meta-learner over the three modality confidence scores can improve on single-modality baselines when no single modality already saturates, motivating multimodal sensing primarily for robustness and redundancy. An end-to-end latency experiment shows that the cloud-uplink leg dominates the budget (79–88%), establishing edge-first inference as a necessary condition for 6G URLLC gains to be observable at the application level. All experiments are conducted over Wi-Fi and MQTT with no 5G or 6G radio, so 6G compatibility is presented as a forward-looking roadmap rather than a tested capability.

## 1. Introduction

Maintenance has evolved from corrective (run-to-failure) to preventive maintenance (PM), and now to predictive maintenance (PdM), which anticipates degradation through continuous monitoring of physical variables. PdM extends asset lifespans, lowers operating costs, and reduces unplanned shutdowns by intervening only when evidence of deterioration justifies it. Relying on advanced sensing, IoT, and machine learning, PdM analyses signals, such as vibration, acoustic, and temperature, to detect early anomalies and trigger proactive interventions. PdM has been reported to reduce costs in rotating machinery, such as AC motors and electric drives, by anticipating bearing and gearbox wear [1,2,3], with multimodal sensor fusion [4] and embedded edge AI [5] identified as central avenues for Industry 5.0.

Traditional PdM systems typically stream raw sensor data to the cloud or high-performance servers for analysis [6]. TinyML, by deploying machine learning models on resource-constrained microcontrollers, drastically reduces this upload, lowering latency, improving privacy, and saving bandwidth [7]. Local edge processing enables decisions in the millisecond range; keeps sensitive vibration, acoustic, and image data on-device; and mitigates security risks [6,8]. Within Industry 5.0, two generations of smart sensors can be distinguished: Sensors 1.0, where transducer, processor, and ML model coexist in an embedded system; and Sensors 2.0, where the inference engine resides inside the sensor module itself, often on a dedicated application-specific integrated circuit (ASIC), delivering only inference results rather than raw samples [9,10].

Wireless technologies have evolved in major decadal milestones. The ITU projects a hundred-fold increase in wireless traffic by 2030 [11]. Sixth-generation (6G) networks are expected to deliver terabit-per-second throughputs, sub-millisecond latencies, massive device densities, and native AI integration [12]. The overall capability framework for IMT-2030 is consolidated in ITU-R M.2160 [13], where standardization in 3GPP is organized into parallel tracks within Release 20 (5G-Advanced and foundational 6G studies), with Release 21 triggering the normative phase [14]. In smart factories, 6G is expected to interconnect huge numbers of intelligent sensors, while edge-AI accelerators in base stations blur the boundary between communication and computation [15,16].

Within this framework, TinyML is complementary to, not a substitute for, 6G: it provides local decision-making in milliseconds, privacy by design, and degraded-mode operation when the link fails. Federated and split-federated learning find their natural places at the intersection of these two planes. To our knowledge, this is the first trimodal vibration–acoustic–thermographic TinyML predictive-maintenance system deployed end-to-end on commercial off-the-shelf hardware, with its anomaly-detection algorithms benchmarked on the actual microcontroller deployment target. The contributions of this work are three-fold: (i) a microcontroller-grade benchmark of five anomaly-detection model variants on real vibration data, evaluated in terms of F1-score, inference time, and both Flash and stack footprints on the actual Cortex-M4F deployment target; (ii) a multimodal TinyML sensing architecture, combining vibration, acoustic and thermographic modalities on commercial off-the-shelf hardware, integrated through an IoT gateway and an AWS IoT cloud back-end, and complemented by two late-fusion analyses an intra-session class-stratified bootstrap and an event-synchronized consistency check providing preliminary evidence of inter-modality complementarity under controlled static-unbalance conditions; and (iii) an end-to-end URLLC latency experiment quantifying how the cloud-uplink leg dominates the budget, motivating edge-first inference as a design invariant for 6G. Theoretically, these contributions show that decision-level fusion of heterogeneous, asynchronous physical signals can be executed on resource-constrained edge nodes rather than in the cloud, and that a low-capacity learned meta-learner can recover faults missed by individual modalities when no single modality saturates; practically, they establish edge-first inference as a necessary condition for a low-latency fault-to-actuation safety loop. Consistent with this scope, the present study is a proof of concept whose primary objective is to demonstrate feasibility on a controlled single-machine testbed, not to provide exhaustive validation across industrial scenarios.

The remainder of the article is organized as follows: Section 2 reviews TinyML, PdM, and 6G; Section 3 presents the architecture; Section 4 describes materials and methods used; Section 5 reports the experimental results; Section 6 discusses the implications of 6G adaptation, URLLC latency, and their limitations; and Section 7 concludes the manuscript.

## 2. Background and Related Work

TinyML denotes the deployment of ML models on tiny, severely resource-constrained devices, typically microcontrollers with CPUs in the tens of MHz, memory in the kilobyte-to-megabyte range, and tight energy budgets [17]. Compact architectures (MobileNets, TinyCNNs) and compression techniques, such as 8-bit (or sub-8-bit) quantization, pruning, and distillation, enable a variety of AI tasks locally with minimal accuracy loss [7,16]. TensorFlow Lite for Microcontrollers [18] and end-to-end platforms, such as Edge Impulse [19], provide the runtime and toolchain. Local processing also yields system-level energy savings, as the radio typically dominates the energy budget of IoT nodes, so well-optimized TinyML sensors can run for months on a coin cell while only transmitting sporadically [16].

PdM instruments machinery with accelerometers, microphones, temperature and pressure sensors, and thermographic cameras to continuously collect data, inferring the health of bearings, motors, and gearboxes from patterns and anomalies in these streams [6,20]. ML models fall into supervised and unsupervised families [6]; data-driven techniques span thresholds, PCA, Isolation Forests, k-NNs, Naïve Bayes, SVM, and deep networks (CNNs, LSTMs) [20,21]. Embedding intelligence directly in the sensors via TinyML overcomes the limitations of cloud-centric architectures, as smart sensors emit only actionable information (anomaly score, class label), reducing network load and enabling sub-second responses, such as automatic shutdowns, without round-tripping to the cloud [20,22]. Distributed inference on low-power microcontrollers has been shown to achieve accuracies above 99% in motor condition monitoring [23]. Recent systematic reviews map TinyML applications, system components, and methodologies for the industrial IoT [24], while incremental and unsupervised TinyML models have been demonstrated for on-device predictive maintenance and anomaly detection, including in extreme industrial environments [25,26].

A recurring finding in the predictive-maintenance literature is that no single sensing modality captures every fault mechanism, which motivates multimodal sensor fusion: the combination of heterogeneous physical signals to broaden fault coverage and improve robustness against missed and false alarms [27,28]. Classical schemes fuse vibration with electrical-current signatures, or add acoustic emissions and temperatures, so that mechanically dominant faults, such as imbalance, misalignment, and bearing wear, and electrically dominant faults, such as broken bars or inter-turn shorts, become jointly observable; for example, Stief et al. fuse acoustic, electrical, and vibration signals from induction motors through a two-stage Bayesian scheme and report fewer missed and false alarms than any single channel [27]. Recent surveys organize these approaches by fusion level, distinguishing data-, feature-, and decision-level fusion and note that decision-level fusion, although discarding some low-level information, is the most tolerant of heterogeneous and asynchronous sources [28]. Deep learning fusion has further pushed multi-sensor diagnosis past 99% accuracy on benchmark machinery by learning joint representations across channels [29]. In particular, vibration–acoustic and broader multimodal fusion based on convolutional and attention networks, feature-level fusion, and noise-robust schemes has been reported to improve bearing- and motor-fault diagnosis over single-sensor baselines [30,31,32,33,34,35]. The architecture proposed here follows this line but differs in two respects: fusion is performed at the decision level directly on resource-constrained edge nodes rather than in the cloud; and it combines vibration, acoustic, and thermographic evidence, extending the vibration–acoustic pairing previously explored for embedded fault detection [4].

Infrared thermography (IRT) is increasingly used for fault diagnosis on rotating machinery because it is non-contact, captures heat generated by friction and electrical losses, and is sensitive to fault mechanisms that vibration alone may miss. Janssens et al. showed that thermal-image features classify a range of bearing conditions and, notably, reveal rotor imbalance—the fault mechanism targeted in this work [36]. Subsequent studies extended IRT to stator and winding faults in induction motors using histogram- and texture-based thermal features with conventional classifiers [37], while more recent work has coupled IRT with convolutional networks to learn the spatial thermal signatures of faults automatically. These results establish IRT as a mature complementary modality for rotating-machine diagnosis and motivate its inclusion, in a TinyML form, alongside vibration and acoustic sensing in the present architecture. An additional argument for adopting thermography as the third modality, rather than as an electrical signature, such as for motor current signature analysis (MCSA), is generality, as current-based diagnosis presupposes an electrically driven machine whose supply current can be measured, while IRT senses the thermal manifestation of friction, load, and unbalance phenomena and is therefore equally applicable to non-electrical rotating equipment—steam turbines, internal-combustion engines, and engine-driven generator sets, such as gearboxes and pumps—in which no drive current exists as a measurable operating parameter [38,39]. The vibration–acoustic–thermographic triad adopted here therefore remains portable to machinery beyond electric motor-driven equipment.

ITU-R M.2160 [13] reorganizes the 5G service space (eMBB, URLLC, mMTC) into six IMT-2030 usage scenarios: three evolved uses (Immersive Communication; Hyper-Reliable Low-Latency Communication, or HRLLC; and Massive Communication) and three new uses (Ubiquitous Connectivity; Integrated AI and Communication; and Integrated Sensing and Communication, or ISAC). Aggregate targets include peak rates near 1 Tb/s, user-plane latencies down to 0.1 ms in HRLLC, reliability above 1 − 10^−7^, and densities up to 10^6^–10^7^ devices/km^2^ [13]. Enabling technologies include sub-THz bands, the upper mid-band, ultra-massive MIMOs, reconfigurable intelligent surfaces (RISs), edge-oriented architectures, and an AI-native air interface, first prototyped in 5G-Advanced via TR 38.843 [40]. Comprehensive surveys of 6G further detail these requirements and applications, together with the associated security challenges [41]. For industrial environments, the path to 6G starts not from the public mobile network but from private and deterministic solutions that 3GPP developed for vertical industries: stand-alone non-public networks (SNPNs), which run a fully independent private 5G core; public-network-integrated non-public networks (PNI-NPNs), which host a dedicated industrial domain over a shared operator infrastructure; and 5G-LAN, which emulates local Ethernet-style connectivity among plant devices. These are combined with IEEE Time-Sensitive Networking (TSN), which adds bounded latency and clock synchronization for real-time control loops, as specified in TS 23.501 and TR 22.821 [42,43], with cyber–physical service requirements in TS 22.104 and TS 22.261 [44,45]. Integrated Sensing and Communication (ISAC) additionally allows the network itself to act as a distributed sensor, reusing the communication waveform to infer presence, motion, and distance of objects in its environment [46,47].

## 3. System Architecture and Key System Components

**Figure 1:**
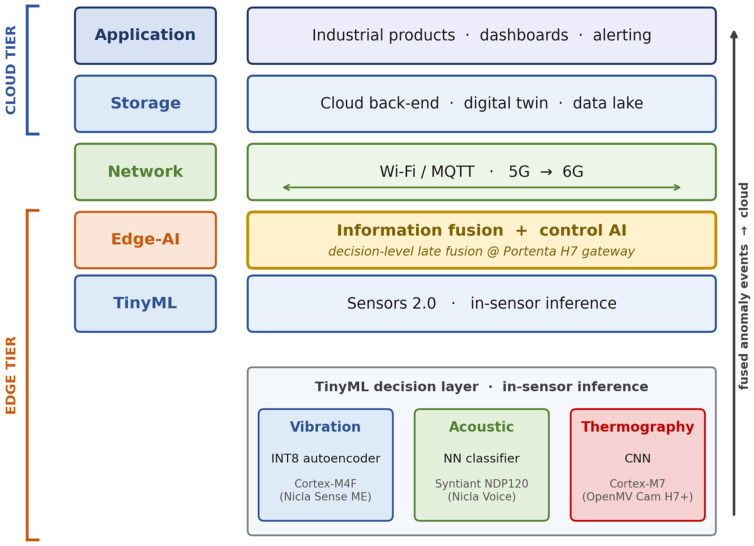
Layered TinyML architecture proposed for predictive maintenance. The bottom TinyML layer hosts smart sensor nodes (Sensors 2.0) running local inference; the edge-AI layer consolidates the per-modality results at the Portenta H7 (Arduino S.r.l., Monza, Italy) gateway, where the decision-level information fusion is designed to execute (in the present prototype, the fusion stage is evaluated offline; Section 4.5, Section 5.4 and Section 5.5); the network layer covers current Wi-Fi/5G and the path toward 6G; and the storage and application layers host the cloud back-end, the digital twin, and the industrial applications fed by the fused anomaly events.

**Figure 2:**
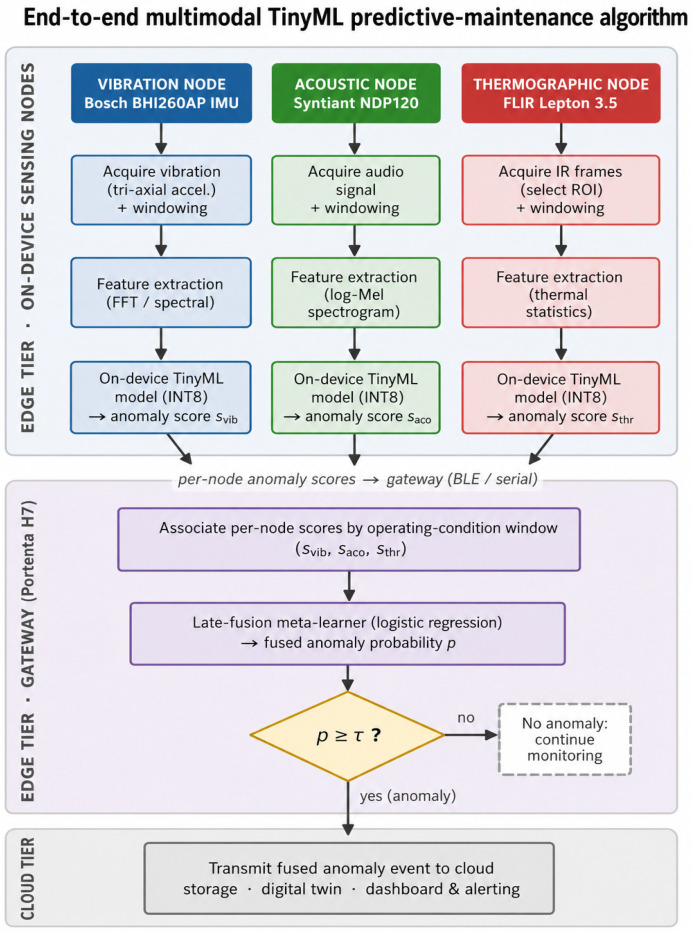
End-to-end flow of the proposed multimodal TinyML predictive-maintenance algorithm. Each sensing node (vibration, acoustic, thermographic) acquires and windows its signal, extracts modality-specific features, and runs an on-device TinyML model (INT8 for the vibration and acoustic nodes; FP32 for the thermographic CNN) that outputs a per-node anomaly score. In the target design, the scores are transmitted to the Portenta H7 gateway, which associates them by their operating-condition window and applies the late-fusion logistic-regression meta-learner to produce a fused anomaly probability; a fused anomaly event is then forwarded to the cloud (storage, digital twin, dashboard, and alerting) only when this probability exceeds the decision threshold, otherwise the system continues monitoring at the edge. In the present prototype, the fusion stage is evaluated offline (Section 4.5, Section 5.4 and Section 5.5).

The proposed architecture (Figure 1) emphasizes local processing at the sensor nodes, with an IoT network layer (and future 6G connectivity) tying the whole system together. The goal is to perform most of the filtering and inference at the edge itself, leaving the network to focus on coordination, control, higher-level analytics, and model updates. Figure 2 summarizes the end-to-end algorithm executed across the sensing nodes, gateways, and the cloud.

### 3.1. Implemented Hardware Architecture

The specific hardware specification used in each modality is summarized in Table 1.

Each smart node carries a microcontroller capable of running ML inference, in line with the Sensors 2.0 paradigm [9,10]. A predictive-maintenance prototype was developed to monitor a laboratory rotating-machine testbed, targeting three failure modes: vibration anomalies, motor acoustics indicative of incipient damage (e.g., unbalance), and thermal signatures associated with imbalance.

**Vibration:** A smart node is mounted on the motor casing. The integrated Bosch BHI260AP (Bosch Sensortec GmbH, Reutlingen, Germany) triaxial MEMS accelerometer exposes preprocessed acceleration vectors via I^2^C to the host MCU of the Nicla Sense ME, an nRF52832 (Nordic Semiconductor, Trondheim, Norway; ARM Cortex-M4F at 64 MHz, FPv4-SP, 512 KB Flash/64 KB SRAM). The TinyML anomaly-detection models reported in Section 5.1 are deployed on this Cortex-M4F host and not on the Fuser2 core internal to the BHI260AP.

**Acoustic:** An acoustic node is placed next to the motor. A neural decision coprocessor extracts the acoustic features (log-Mel bins) internally, and an in-sensor model classifies them.

**Figure 3:**
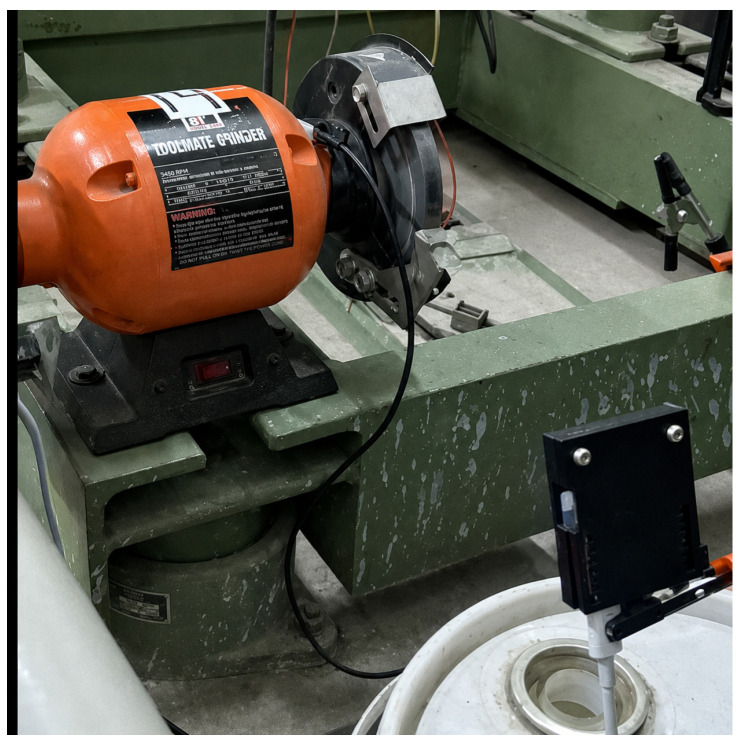
Smart sensor nodes deployed on the laboratory test motor. The vibration node (with the triaxial MEMS accelerometer) is mounted on the motor housing; the acoustic node faces the motor at a short distance; and the thermographic node is positioned in front of the motor. The test disk (Section 4.1) is mounted on the rotating shaft to induce calibrated unbalance.

**Vision:** An embedded thermographic camera, coupled to an infrared microbolometer, is placed in front of the motor to capture heat maps (thermograms) of the casing, in which each pixel encodes a surface-temperature value (Figure 3).

### 3.2. Network and Data Integration

**Figure 4:**
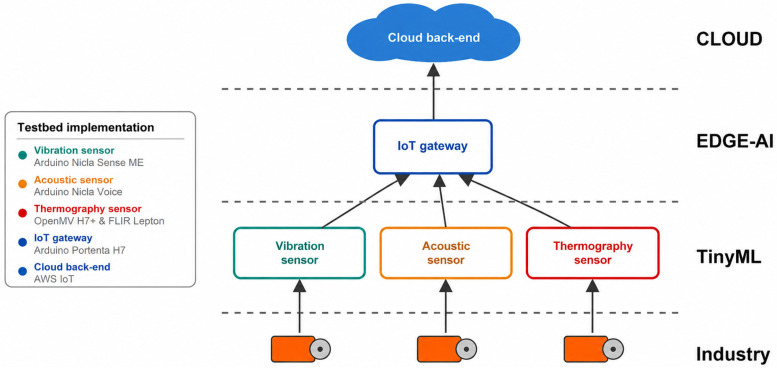
End-to-end network integration. The three smart sensor nodes (Nicla Sense ME for vibration, Nicla Voice for acoustic, OpenMV Cam H7+ with FLIR Lepton for thermography) execute TinyML inference locally; the Arduino Portenta H7 gateway aggregates their results and forwards them via Wi-Fi/MQTT to AWS IoT Core, where they are stored, visualized, and dispatched as alerts.

The three sensor nodes feed their TinyML models locally and raise alarms when appropriate. They are connected through the Eslov connector, an I^2^C-based serial interface from the Arduino Pro family (not an industrial fieldbus, such as Modbus or PROFIBUS), to an IoT gateway that manages the devices and acts as a Wi-Fi/MQTT bridge to a cloud platform deployed on AWS. The Eslov link supports up to 3.4 Mbit/s in I^2^C High-Speed mode. The BHI260AP runs the BSX virtual-sensor fusion pipeline internally and exposes preprocessed three-axis acceleration vectors to its host MCU at a configurable cadence; in this work, the application-layer cadence is set to 10 Hz of feature vectors, corresponding to approximately 480 bit/s of raw payload, many orders of magnitude below the I^2^C capacity (Figure 4).

### 3.3. Cloud Infrastructure

**Figure 5:**
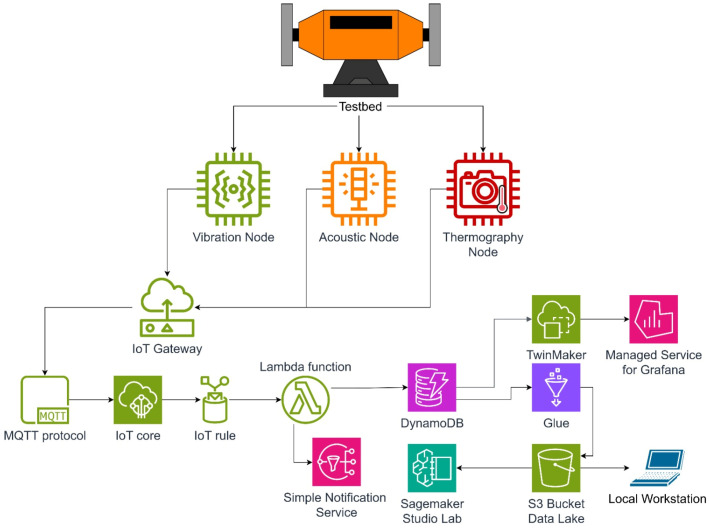
Cloud architecture deployed on AWS. Inbound MQTT messages from the IoT gateway are routed by an IoT Core into a Lambda function that decodes, persists in DynamoDB, and dispatches the payload. Detected anomalies trigger SNS notifications. A data lake on S3, fed by Glue, supports offline training in SageMaker, while TwinMaker and the Grafana-based dashboard visualize the digital twin.

**Figure 6:**
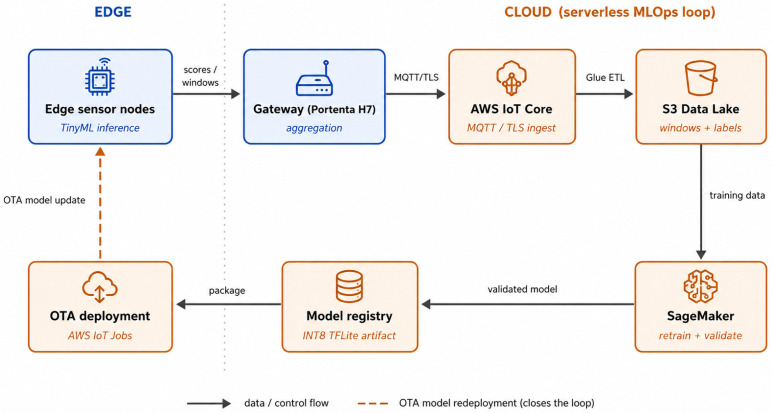
Closed-loop model-iteration workflow linking edge inference and the cloud MLOps pipeline. Telemetry, namely anomaly scores and labeled vibration windows, flows from the TinyML edge nodes through the Portenta H7 gateway and AWS IoT Core into the S3 data lake; SageMaker then retrains and validates the updated model, which is quantized to an INT8 TFLite artifact, registered, and pushed back to the edge nodes through an over-the-air (OTA) update (dashed arrow), closing the iteration loop.

Amazon Web Services (AWS; Seattle, WA, USA) are a widely adopted public-cloud provider that offers, on demand, a broad collection of managed services for storage, data processing, machine learning, and IoT connectivity. The cloud platform of this work was developed on AWS under the serverless paradigm, in which the user specifies only which services are used and how they interact, while AWS manages the underlying infrastructure [8,48]. The platform retains data from the vibration and acoustic nodes together with the inference results from the thermographic images; trains, validates, and deploys ML models; and serves a Grafana-based dashboard for real-time visualization. Figure 5 shows the platform architecture, detailing the main services and their interactions: the IoT Core for MQTT and certificate-based device management, Lambda for event-driven processing, DynamoDB for structured records, S3 for the data lake [49], SageMaker for centralized training [50], TwinMaker for the digital twin, Glue for ETL, and SNS for alerting [51]. Beyond serving inference results, this cloud tier closes the model-iteration loop, where SageMaker periodically retrains and validates the models on the accumulated data, and the updated, quantized artifacts are redeployed to the edge nodes over the air, as detailed in Figure 6.

## 4. Materials and Methods

### 4.1. Rotating Machine Testbed and Unbalance Protocol

In this section, we describe the experimental testbed and the controlled unbalance protocol used to generate the labeled vibration dataset that underpins the anomaly-detection experiments of Section 5. A central difficulty in predictive-maintenance research is obtaining fault data, as healthy operation is abundant, but faults are rare and costly to reproduce on production machinery. To address this, we designed and implemented a realistic testbed built around a rotating machine, incorporating a mechanism to artificially and repeatably induce abnormal operation and controlled static unbalance, so that both healthy and faulty states can be recorded on demand and at different severities.

Static unbalance occurs when the principal axis of inertia of the rotor is displaced parallel to the axis of rotation, generating a mass eccentricity that produces a non-zero net centrifugal force. The vibration amplitude is directly proportional to the unbalancing mass and its eccentricity, so adding mass at a given angular position deliberately increases vibration and generates fault signatures, while removing mass (or adding it at the opposite side) reduces vibration toward the balanced reference.

**Figure 7:**
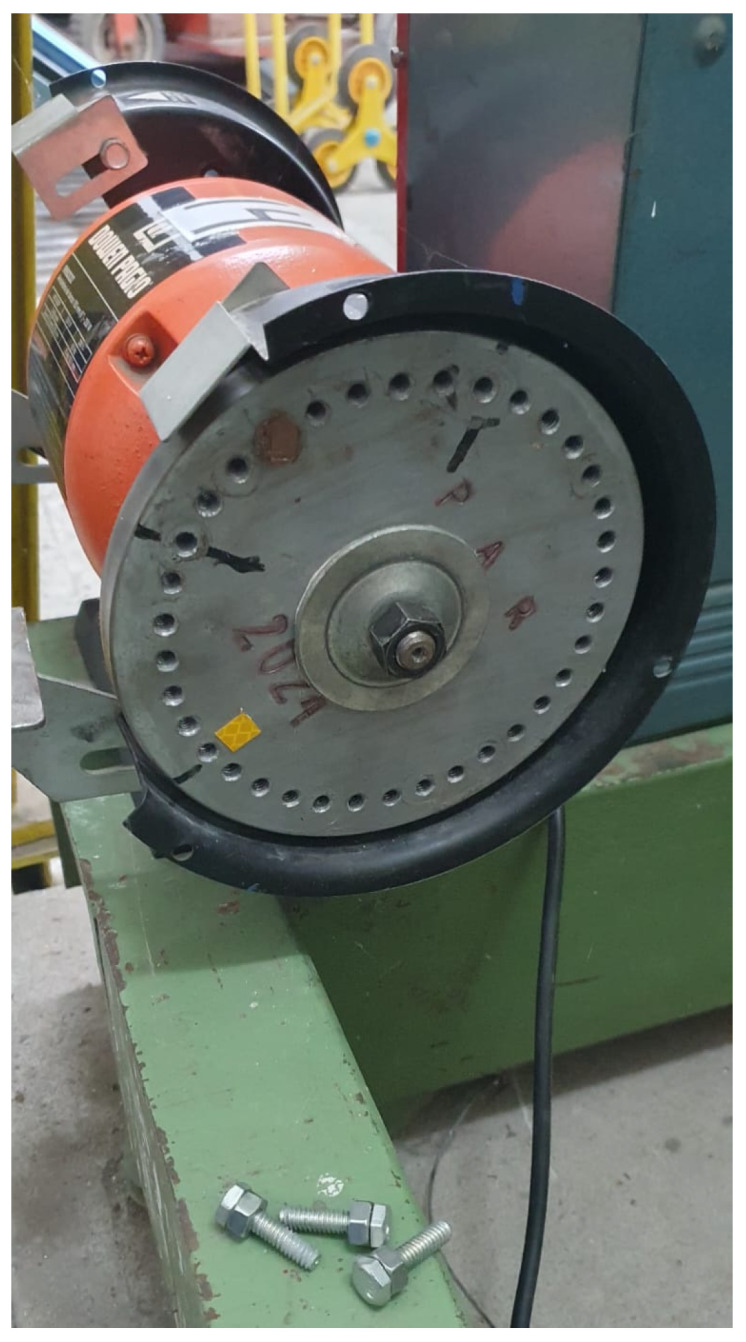
Balancing disk used in the static-unbalance protocol (200 mm diameter, 10 mm thickness, 36 M12 holes every 10°). M12 screws of calibrated mass are inserted at controlled angular positions to induce or compensate unbalance, enabling reproducible generation of vibration signatures across a range of severities.

**Testbed:** A balancing disk, with a 200 mm diameter and 10 mm thick, was designed with 36 M12 threaded holes machined every 10° around the perimeter (Figure 7), allowing a calibrated mass to be placed at any angular position. The disk was mounted on a Dowen Pagio AB150P bench grinder (Barbuy Team S.A., Bell Ville, Argentina; 250 W, 2950 rpm). During data acquisition, the vibration node was bonded directly to the grinder casing so that the triaxial accelerometer was rigidly coupled to the housing, while the acoustic node was positioned approximately 10 cm from the motor to capture airborne sound (Figure 3). The experiment proceeds in two stages: (i) unbalance induction, in which an M12 screw is mounted at a known angular position to introduce a calibrated eccentric mass; and (ii) unbalance correction, in which the magnitude and phase angle measured by the vibration instrument determine the compensation mass placed at the opposite angular position. The mass is then iteratively trimmed and the vibration re-measured until the residual amplitude is minimized. This protocol bidirectionally modulates the vibration level, yielding the dataset used in this work (balanced healthy and unbalanced faulty states with different severities). Table 2 summarizes the resulting measurement dataset across the three sensing modalities. The dataset is deliberately structured around two principal operating states, healthy (balanced) and faulty (unbalanced), with the faulty state reproduced at two discrete severities, instead of around a continuum of naturally evolving intermediate degradation stages. Obtaining such intermediate states through natural wear would require run-to-failure campaigns spanning the service life of the machine elements; rolling bearings are typically dimensioned for L10, rating lives of the order of 10,000–40,000 operating hours [52], which is precisely why bearing-prognostic platforms resort to artificially accelerated degradation rather than natural-life testing [53]. Experiments of that duration are infeasible on the present testbed, so discrete, repeatable unbalance severities are used as controlled proxies for fault progression.

### 4.2. Vibration-Based Anomaly Detection

Vibration patterns depend on equipment-specific parameters, such as rotational speed and mounting [54,55,56]. Obtaining labeled fault data on production lines is impractical; a more useful strategy is to learn the vibration pattern under normal conditions and flag deviations indicative of malfunction, i.e., one-class anomaly detection [57,58], in which the training set contains a single class (healthy operation). Autoencoder reconstruction has proven particularly effective on industrial motors without labeled fault data [5,21].

In this work, ten statistical features are extracted from 6 s windows of the accelerometer signal (60 samples per window at the 10 Hz host cadence of Section 3.2). This feature set avoids expensive preprocessing, such as Fourier transform, and, being frequency independent, reduces the dependency on operating speed. The ten features (Table 3) are standard time-domain descriptors widely used in vibration-based condition monitoring [54,55,56] and recent TinyML-PdM publications [4,5]. With *N* = 60 samples per window, the higher-order moments (skewness and excess kurtosis) are estimated with acceptable variance; substantially shorter windows would render these fourth-order descriptors statistically unreliable. It should be made explicit that the 10 Hz host cadence sets a 5 Hz Nyquist limit, well below the approximately 49 Hz rotational frequency of the bench, so the host-exposed signal does not resolve the 1× unbalance spectral line and no spectral analysis is attempted; this cadence is a hardware constraint of the Nicla node, which cannot be polled faster. The ten time-domain features instead characterize the amplitude and energy-envelope statistics of each 6 s window, which, in our measurements, shift monotonically with unbalance severity; it is on these statistics, rather than on any resolved fault frequency, that anomaly detection relies.

Data are collected from the testbed with the disk in balanced and unbalanced configurations across several severities. Windows of 6 s are extracted contiguously without shuffling between recordings. Healthy condition measurements are partitioned into 60% for training, 20% for validation, and 20% for testing. The healthy test subset is then combined with unbalanced-condition measurements to form the final test partition used for anomaly-detection evaluation. The anomaly threshold is set at the 95th percentile of the reconstruction-error or projection-score distribution computed on the healthy training windows, and is never tuned on the test partition.

Five anomaly-detection model variants, corresponding to four distinct algorithms, were evaluated: Principal Component Analysis (PCA); a fully connected autoencoder evaluated, both in FP32 and INT8 (Q8INT) form via post-training quantization; One-Class SVM; and an Isolation Forest. Autoencoder hyperparameters were tuned via Random Search over 10 configurations (encoder/decoder depths ∈ {1,2,3}, layer widths 8–64, bottleneck 2–8, Adam, MSE loss, early stopping); consequently, the winning architecture is 32 → 8 → 8 → 16 → 10 with ReLU hidden layers and linear output. The OC-SVM uses an RBF kernel; the Isolation Forest uses 100 trees (the scikit-learn default) in the offline benchmark, while the on-target implementation executes a distilled ensemble of 6 trees of maximum depth 8, whose latency and footprint are the figures reported in Table 4. Features are standardized to zero mean and unit variance using training-only statistics.

The four algorithms share the one-class decision structure introduced above, differing only in how the model of normal behavior and the resulting anomaly score are defined. In each case, a model is fitted exclusively on healthy windows, a scalar anomaly score is computed for each incoming window, and the window is flagged as faulty when this score exceeds the 95th-percentile threshold of the healthy training distribution.

Principal Component Analysis (PCA) retains the principal components required to explain 95% of the variance of the healthy training windows (four components in this dataset) and reconstructs each window from this linear subspace; the per-window mean-squared reconstruction error is the anomaly score, thresholded at its 95th percentile on the healthy training set and consistent with the shared decision rule described above. The fully connected autoencoder generalizes this principle to a non-linear manifold: an encoder–decoder network is trained to minimize the reconstruction error of healthy windows, and the per-window mean-squared reconstruction error is used as the score, which grows for inputs the network was never trained to reproduce. The One-Class SVM maps the feature vectors into a high-dimensional space through an RBF kernel and learns the maximum-margin boundary that separates the healthy data from the origin; the signed distance to this boundary is the decision function, and a window falling on its outer side (negative decision value) is declared anomalous. The Isolation Forest adopts a complementary, density-free view; it builds an ensemble of random binary trees that recursively partition the feature space and exploits the fact that anomalous windows, being few and different, are isolated with fewer splits, so the score is derived from the average path length required to isolate a window, with shorter paths denoting anomalies. This shared score-and-threshold formulation makes the detectors directly comparable in Section 5.1 while spanning the linear-reconstruction, non-linear-reconstruction, boundary-based, and isolation-based families of one-class anomaly detection.

### 4.3. Acoustic Modality and Log-Mel Features

Acoustics are an established source for early fault detection, particularly when vibration sensing is impractical or when normal operation produces apparently anomalous vibration [59,60,61]. Acoustic processing demands more computation than acceleration-based methods, so the acoustic node integrates a neural decision coprocessor (Syntiant NDP120; Syntiant Corp., Irvine, CA, USA) that combines a DSP and an in-sensor neural engine. The firmware pipeline is generated through Arduino Cloud and Edge Impulse (Edge Impulse Inc., San Jose, CA, USA), where healthy and faulty acoustics are captured at 16 kHz mono with the on-board MEMS microphone, uploaded to the Edge Impulse cloud project, processed through the “log-bin” feature extractor specific to the NDP120/200 family [19,62], and the trained classifier is deployed back onto the application-specific integrated circuit (ASIC). The NDP120 is optimized to operate directly on log-Mel filterbank energies (the representation produced just before the DCT in a classical MFCC pipeline), and the “log-bin” block omits that final DCT step. The exact frame length, stride, Mel-filter count, FFT length, and pre-emphasis coefficient used here are listed in Section 5.2 (Table 5).

### 4.4. Thermographic Modality and CNN Classifier

Vibration- and acoustic-emission-based PdM is sensitive to environmental noise, as simultaneous operation of nearby machinery, operator activity, ambient conditions, and EMI degrade the signal-to-noise ratio and may mask incipient faults [54,63]. Infrared thermography (IRT) is non-contact and non-invasive, making it a useful complement [64]. Traditional industrial thermal-vision systems stream massive radiometric flows to cloud servers, with latency, cybersecurity, and bandwidth costs that the TinyML paradigm directly addresses [22,64].

**Figure 8:**
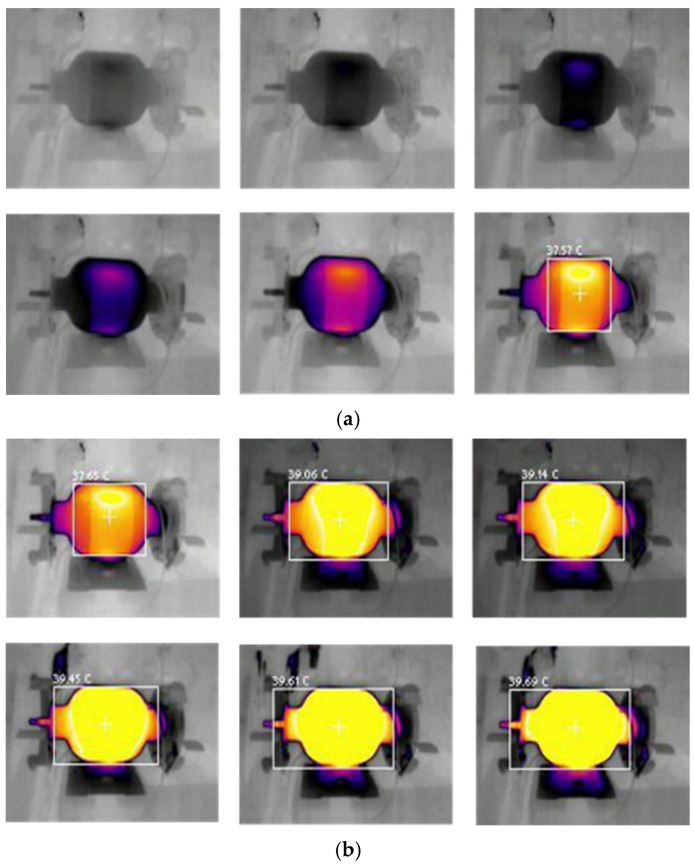
Sample 96 × 96 px thermographic frames captured by the OpenMV Cam H7+ coupled to the FLIR Lepton (Teledyne FLIR LLC, Wilsonville, OR, USA) microbolometer. (**a**) Frames acquired under nominal (balanced) operation, showing a stable thermal pattern. (**b**) Frames acquired under induced static unbalance, showing the increased surface temperature associated with friction at the bearings and the vibrating support.

MP4 videos of the laboratory test motor were captured with the embedded thermographic camera, with one under nominal (balanced) operation and a second after the disk was unbalanced on the same day with the same setup; a Python script (Python 3.13.5) using the OpenCV library (opencv-python 4.13.0) extracted individual frames. Each frame was resized to 96 × 96 px, three RGB channels, normalized to [0, 1] and labeled 0 (normal) or 1 (fault); representative frames of both classes are shown in Figure 8. A lightweight convolutional neural network maps each 96 × 96 × 3 frame to a single fault-probability output, where three convolutional blocks with 16, 32, and 32 filters, each using 3 × 3 kernels with ReLU activation and followed by 2 × 2 max-pooling, are succeeded by a flatten layer, a 64-unit dense layer with ReLU activation, and a one-unit dense layer with sigmoid activation; the model has approximately 219 k parameters and is deployed in 32-bit floating-point precision. A binary normal/fault formulation is adopted because only the two operating conditions recorded in this study are available and because this modality contributes a fault-presence score to the late-fusion stage rather than grading fault severity. As discussed in Section 4.1, populating intermediate severity grades with naturally evolved degradation data would require run-to-failure tests on the scale of the bearing service life [52,53], which is infeasible for the present testbed. Training used the Adam optimizer with binary cross-entropy loss, dropout regularization (rates 0.5 and 0.3), and early stopping with restoration of the best weights (Table 6); the trained model is then converted to TensorFlow Lite (32-bit floating point) for embedded execution on the OpenMV Cam H7+ (OpenMV LLC, Atlanta, GA, USA).

Because each class is represented by a single continuous video, the train/test split is enforced at the frame level rather than at the video or session level, and adjacent frames are highly temporally correlated; the implications for the reported metrics are analyzed in Section 5.3 and Section 6.4. Robustness-oriented neural architectures developed for infrared thermography, such as attention-based networks [65], are natural candidates to mitigate this sensitivity and are identified as directions for future work.

### 4.5. Multimodal Late-Fusion Method

The three modalities are combined at the decision level, after each per-modality model has produced its own confidence score, rather than at the raw-signal or feature level. This late-fusion choice is dictated by the heterogeneity of the prototype, as the vibration, acoustic, and thermographic nodes run independent in-sensor pipelines with different sampling rates, feature spaces and inference cadences, so a common feature tensor cannot be formed at the edge. Four fusion strategies are evaluated and contrasted. The three rule-based strategies operate directly on the binary per-modality decisions: logical OR flags a fault when any modality fires, maximizing recall at the cost of false alarms; logical AND flags a fault only when all three modalities agree, maximizing precision at the cost of missed detections; and majority vote flags a fault when at least two of the three modalities agree, balancing the two extremes. The fourth strategy is a learned meta-learner, where a logistic-regression classifier takes the three continuous modality confidence scores as input features and is trained, with five-fold stratified cross-validation, to predict the fault label, so that the relative weight of each modality is estimated from data rather than fixed a priori.

Because the three nodes acquire data through independent pipelines, the modality scores are not aligned at the level of individual events, and a strictly event-synchronized test set is therefore not available for this prototype. To evaluate fusion under this constraint, a class-stratified bootstrap protocol is adopted. At each of 1000 iterations, 150 synthetic test events are assembled by drawing, with replacement, 50 normal-operation events and 100 faulty-operation events spanning fault severities from mild to severe; each synthetic event aggregates one score per modality drawn from the corresponding class and severity pool. The procedure preserves the true class label of every drawn score and controls the relative contribution of the different fault severities within the faulty class, yielding a distribution of performance metrics from which means and 95% confidence intervals are computed. This design provides a statistically meaningful estimate of marginal inter-modality complementarity while making explicit that it does not rely on genuinely co-occurring observations; the resulting metrics are reported in Section 5.4.

Moving from this offline analysis to an online in-sensor fusion pipeline requires explicit temporal alignment of the three decision streams, which the current prototype does not yet implement. The intended synchronization scheme places the alignment at the Arduino Portenta H7 gateway, which already aggregates the three nodes over the Eslov/I^2^C link. Each per-modality decision is time-stamped against a single reference clock distributed over the network, using Network Time Protocol (NTP) for soft real-time deployments and IEEE 1588 Precision Time Protocol (PTP) or the bounded-latency services of IEEE Time-Sensitive Networking (TSN) where deterministic alignment is required. The gateway then buffers the time-stamped decisions and groups them into a common sliding observation window whose width is set by the slowest modality, namely the thermographic frame rate, so that a single fused decision is emitted per window from genuinely co-occurring per-modality scores. This gateway-centric design keeps the in-sensor nodes unchanged and confines the synchronization logic to one device; its experimental validation on event-synchronized cross-session data is left to future work.

## 5. Results

### 5.1. Vibration Anomaly-Detection Benchmark

The five anomaly-detection algorithms were evaluated by F1-score, accuracy, and inference time. Two complementary inference-time measurements are reported using a per-sample latency on a reference laptop (AMD Ryzen 7 260, 3.80 GHz, 32 GB RAM), and a single-inference measurement on the target Cortex-M4F host (nRF52832 of the Nicla Sense ME, not the BHI260AP Fuser2 core); the latter of which is the median of 16,605 single-shot calls instrumented with micros() in the Arduino runtime (Arduino Mbed OS Nicla Boards core, version 4.6.0). Although the BHI260AP exposes an in-sensor compute fabric, its toolchain is restricted, whereas the nRF52832 runs standard Arduino-mbed firmware and supports the same C/C++ deployment path that would be used in production. Table 4 reports the full benchmark.

PCA, despite being the lightest candidate (13 µs and 132 B on the MCU), achieves only F1 = 0.6689 with accuracy 0.559. This is the expected consequence of a linear-projection detector applied to a fault class whose feature-space signature partially overlaps with the normal regime, as the moderate-unbalance windows are reconstructed almost as accurately as healthy ones (mean reconstruction error 0.089 against a threshold of 0.124), so 79% of them fall below the 95th-percentile threshold and are mis-flagged as normal, while the severe class (mean error 1.09) is flagged in 98.7% of windows. PCA is therefore retained as a reference baseline but is not a viable deployment candidate. The four non-linear models all reach F1 above 0.98, led by the Isolation Forest (0.9878 [0.978, 0.997]), followed closely by OC-SVM (0.9831) and the two autoencoder exports (0.9807 [0.971, 0.990], identical decisions in FP32 and Q8INT), all with perfect fault recall. On the Cortex-M4F, the ranking is PCA (13 µs) < Isolation Forest (32 µs) < FC autoencoder Q8INT (254 µs) < FC autoencoder FP32 (293 µs) ≪ OC-SVM (7057 µs). The non-uniform slowdown factors have a clear architectural origin: Isolation Forest is a sequence of integer comparisons through a binary tree, natively supported by the M4F pipeline; the INT8 autoencoder exploits the CMSIS-NN integer kernels, which pack four 8-bit operations per 32-bit MAC, where the FP32 variant has to issue one floating-point MAC per coefficient; the OC-SVM evaluates a kernel against 28 support vectors per inference, translating into ~280 dot products of floating-point arithmetic per call without SIMD acceleration. This is precisely the kind of cross-platform reordering that justifies characterizing inference on the deployment target rather than extrapolating from a laptop figure.

Post-training quantization yields a 13% reduction in inference time, a 9% reduction in Flash, and an 8% reduction in stack with bit-identical detection decisions and therefore no F1 degradation, identifying the INT8-quantized autoencoder as the recommended deployment candidate; it remains within 0.007 F1 of the best-performing candidate while occupying 2.4× less Flash, and its continuous reconstruction-error score feeds the late-fusion stage of Section 4.5 directly, unlike the thresholded tree ensemble, with a per-inference latency approximately 394× below the 100 ms inter-sample period of the I^2^C accelerometer stream, and generalizes correctly to faults whose feature-space distribution overlaps with normal. All four working candidates fall well within the 64 KB SRAM/512 KB Flash budget of the nRF52832 and support future co-deployment of additional modalities. The F1 values obtained with the four working algorithms are competitive with those recently reported in [7] for TinyML-based PdM on motors and with the 99% accuracies reported above for distributed on-device motor condition monitoring [3], while additionally providing on-target inference latency and Flash/stack footprints, figures that prior works generally omit. Finally, the corrected protocol enables a controlled cross-session check of this modality, where transferring the frozen session 1 autoencoder to a second acquisition with the remounted sensor preserves perfect fault recall but flags 99% of healthy windows, and re-estimating only the input scaler and decision threshold on a healthy baseline of the new installation restores F1 = 0.9751 [0.961, 0.987]; the full analysis is reported in Section 6.4.

### 5.2. Acoustic Classifier

**Figure 9:**
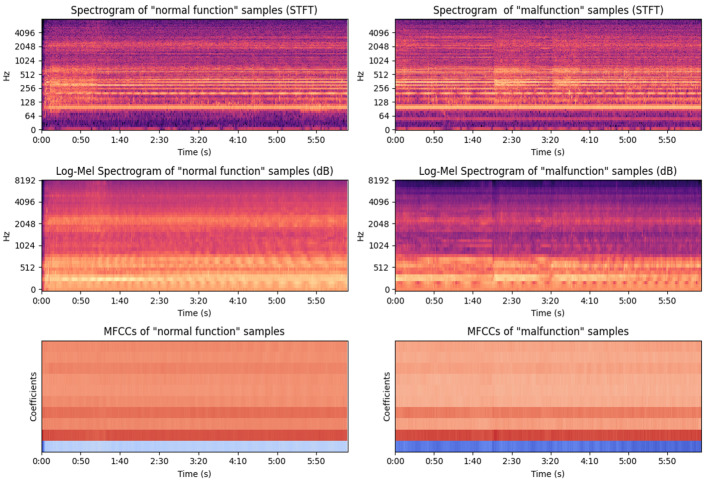
Acoustic feature representations for healthy and faulty motor samples. **Top**: Short-time Fourier transform (STFT) spectrogram. **Middle**: Log-Mel spectrogram with 40 Mel bands, used as the on-chip feature representation for the NDP120 deployment. **Bottom**: First 10 MFCC coefficients derived from the same log-Mel spectrum, shown as an analytical complement; the NDP120 “log-bin” extractor omits the DCT and consumes the log-Mel bins directly.

Acoustic samples of the test machine under healthy and faulty operation were recorded through the integrated Nicla Voice microphone (16 kHz mono, 16-bit) and uploaded to Edge Impulse. The “log-bin” feature extractor configured for the NDP120/200 family computes log-Mel filterbank energies over a frame length of 32 ms and a stride of 24 ms (8 ms overlap), with 40 Mel filters, an FFT length of 512 samples and a pre-emphasis coefficient of 0.96875. The features are fed to a compact two-dimensional convolutional classifier deployed onto the NDP120 ASIC. The 1600 per-window log-Mel energies (40 Mel bins × 40 frames) are reshaped into a 40 × 40 single-channel map and processed by two convolutional blocks, each with eight 3 × 3 filters, ReLU activation and 2 × 2 max-pooling, followed by a dropout layer (rate 0.25), a flatten layer and a two-unit softmax output (normal/abnormal). The network was trained in Edge Impulse for 20 epochs with the Adam optimizer (learning rate 0.0005) and categorical cross-entropy loss, without data augmentation, and post-training quantized to 8-bit integer (int8) for in-sensor execution. In faulty samples, additional frequency bands carrying significant energy appear in the log-Mel spectrogram (Figure 9) that are imperceptible in the healthy baseline.

Table 5 reports the quantitative performance. The int8-quantized model deployed to the Syntiant Core 2 achieves perfect class separation on the validation set in this experimental configuration, with F1, accuracy, precision, recall and AUC equal to 1.00 and zero false positives or negatives. The dataset comprises 19 min 48 s of acoustic captured during a single laboratory session, with the Edge Impulse train/test split of 65%/35% by duration. The model occupies 1.375 KB of parameter memory (0.2% of the 640 KB budget) and consumes an estimated 5.55 µJ per inference at 0.9 V. The Core 2 is a streaming in-sensor accelerator: it produces one classification per frame and is latency-bounded by the 24 ms frame stride (~42 inferences/s) and not by ASIC throughput. This single-session structure means that the training and test segments are cut from a single continuous recording and therefore share the same ambient acoustic environment, microphone placement and motor warm-up state; the F1 = 1.00 demonstrates intra-session separability of the recorded acoustic data and must not be read as generalization performance. Cross-session validation is required to establish field performance and is left to future work, a limitation revisited in Section 6.4.

### 5.3. Thermographic CNN

**Figure 10:**
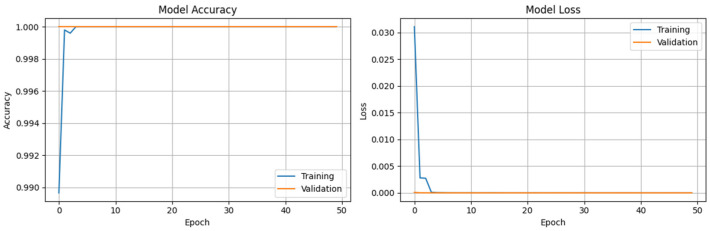
Training and validation curves of the binary thermographic CNN classifier (healthy vs. faulty motor) over 50 epochs. **Left**: Classification accuracy. **Right**: Binary cross-entropy loss. As discussed in Section 6.4, these curves reflect intra-session separability of frames sampled from two single-session videos with a frame-level train/test split and not cross-session generalization.

The architecture and training configuration of the binary thermographic CNN are summarized in Table 6. Figure 10 reports the training and validation curves of the binary thermographic CNN. Accuracy reaches values close to 100% within the first few epochs on both partitions, and the binary cross-entropy loss falls close to zero and remains there. As anticipated in Section 4.4 and discussed in Section 6.4, these curves should be interpreted as evidence that the CNN cleanly separates the frames of the two videos available for this study (one continuous video of the balanced motor and one of the unbalanced motor, both captured in the same session) rather than as evidence of cross-session generalization; with the train/test split applied at the frame level over two single videos, the temporal correlation between adjacent frames is the dominant signal the network can exploit. For the embedded camera, the trained model is loaded from on-board memory; each captured frame is preprocessed to 96 × 96 px and classified on-device at ~2 frames per second (~462 ms per frame; Table 6), with sliding statistics of the fault rate displayed on-screen with periodic console reports. Moreover, because both videos were recorded in a single session, the temporal correlation between adjacent frames and the progressive motor warm-up are confounded with the fault signature and cannot be ruled out under the present single-session de-sign; the near-100% accuracy therefore reflects intra-session separability rather than cross-session generalization.

### 5.4. Multimodal Late Fusion

Section 5.1, Section 5.2 and Section 5.3 evaluate each modality in isolation. To address whether the three modalities provide complementary or merely redundant evidence, a late-fusion experiment was carried out on three recorded sessions of the same motor: one balanced (N) and two unbalanced (moderate A and severe MA). The three modalities were captured under identical operating conditions but with each sensor operating on its own independent acquisition pipeline; therefore, strict event-level synchronization is not available, and the experimental design accommodates this with a class-stratified bootstrap protocol.

**Figure 11:**
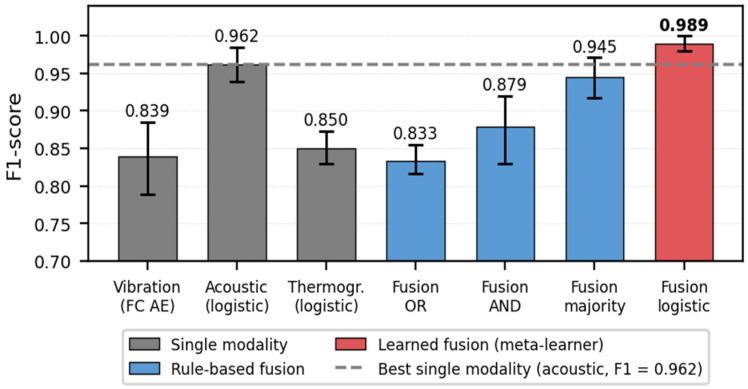
Multimodal late-fusion F1-scores for the three single-modality baselines (vibration, acoustic, thermographic) and four late-fusion strategies (logical OR, logical AND, majority vote, and logistic regression on confidence scores trained via five-fold stratified cross-validation). Bars show the mean F1 over 1000 bootstrap iterations of 150 events per iteration (50 normal, 100 fault); error bars show 95% confidence intervals. The dashed horizontal line marks the best single-modality baseline (acoustic, F1 = 0.962). The logistic-regression meta-learner (rightmost bar) is the only fusion strategy whose 95% CI lies entirely above the best single-modality baseline.

The four late-fusion strategies and the class-stratified bootstrap protocol used to assemble the synthetic test events are defined in Section 4.5. Table 7 reports the resulting metrics as bootstrap means with 95% confidence intervals, and Figure 11 visualizes the corresponding F1-scores. Notably, for the acoustic and thermographic modalities, the fusion is fed by offline scores re-derived from features (logistic regression on log-Mel and texture features, respectively) rather than by the in-sensor decisions of the NDP120 ASIC and the embedded CNN; therefore, the fusion is an offline analysis and not the in-sensor decision pipeline.

Several observations follow: First, the vibration-only FC autoencoder reaches F1 = 0.839 [0.788, 0.885] with precision 0.89 and recall 0.79; a per-source breakdown shows that the limitation is concentrated on mild-to-moderate fault conditions, of which only 59% is correctly detected, while 100% of severe fault events and 81% of normal-operation events are correctly classified, the expected behavior of a one-class autoencoder on a fault class whose feature-space statistics partially overlap with the normal regime. Second, the acoustic baseline reaches F1 = 0.962 [0.939, 0.985] with perfect recall but 7–8 false positives per 50 normal events, reflecting the higher sensitivity of log-Mel features to background-noise variability. Third, the thermographic baseline reaches F1 = 0.850 [0.830, 0.873]; its lower precision reflects the difficulty of separating thermal patterns of the unbalanced motor from normal warm-up patterns when only frame-level texture features are available. Fourth, the rule-based fusion strategies show the expected pattern: OR amplifies the false-positive rate of the most permissive modality (F1 = 0.833); AND inherits the missed-detection rate of vibration on mild-to-moderate fault conditions (F1 = 0.879 with recall 0.79); and majority vote averages out the precisions and lifts F1 to 0.945 [0.917, 0.971], close to the best single modality. Fifth, the logistic-regression meta-learner reaches F1 = 0.989 [0.980, 1.000] with accuracy 0.985, precision 0.979, and a perfect recall, improving on every single-modality baseline and on every rule-based fusion strategy in the mean and across the 95% confidence interval. This additive gain is, however, a conditional estimate, as it is obtained in the regime probed by this bootstrap, in which the offline acoustic baseline is not saturated (F1 = 0.962) and therefore leaves headroom for complementary evidence; it should be read as a secondary indicator rather than as the central claim of the work. When one modality already separates the classes perfectly, as the acoustic channel does on the synchronized acquisition of Section 5.5, no additive gain over the best single modality is possible by construction, and the value of the multimodal design is then redundancy and robustness rather than an additive score. The confusion matrix averages 47.8 TN and 2.2 FP out of 50 normal-operation events, and 100 TP with no FN out of 100 faulty-operation events. Taken together, these results provide preliminary evidence of inter-modality complementarity under intra-session conditions; because the three modalities were not synchronized at the event level, the fusion operates over a class-stratified bootstrap of independently drawn modality scores rather than over genuinely co-occurring observations, so the complementarity holds in a marginal statistical sense. An event-synchronized fusion on a second, simultaneously recorded campaign is now reported in Section 5.5, which confirms the consistency of the modality contributions on genuinely co-occurring observations; cross-session and cross-machine confirmation remains for future work. The practical advantage of the multimodal design most relevant for deployment is that it recovers the ~41% of moderate-unbalance faults that no single sensor reliably detects, while keeping false alarms at about two per fifty normal events (precision 0.979, perfect recall).

### 5.5. Time-Synchronized Late-Fusion Analysis

To address the event-synchronization limitation of the bootstrap analysis in Section 5.4, a second session was recorded on a different day on the same test bench, in which the three modalities were acquired simultaneously for each operating condition (balanced (N), moderate unbalance (A), and severe unbalance (MA)). Each modality was aligned on a common wall clock: vibration windows from the acquisition timestamp; thermographic frames from the burned-in overlay clock; and the acoustic stream anchored to the motor-on instant, since each recording begins with the motor already running. Co-occurring events were then assembled at a 6 s cadence over the overlapping operating interval of every condition, yielding 323 genuinely simultaneous events (148 healthy and 175 faulty). These 6 s events are non-overlapping, as consecutive events are drawn from disjoint 6 s segments (stride equal to the window length), so no sample is shared between events. This ensures that the frame-to-frame temporal correlation noted for the single-session benchmarks (Section 6.4) does not inflate the scores on the synchronized set, and that the 323 events are statistically independent observation windows rather than a sliding-window expansion of a shorter recording. This provides the event-synchronized fusion that Section 5.4 identified as necessary for a physically grounded complementarity claim.

**Figure 12:**
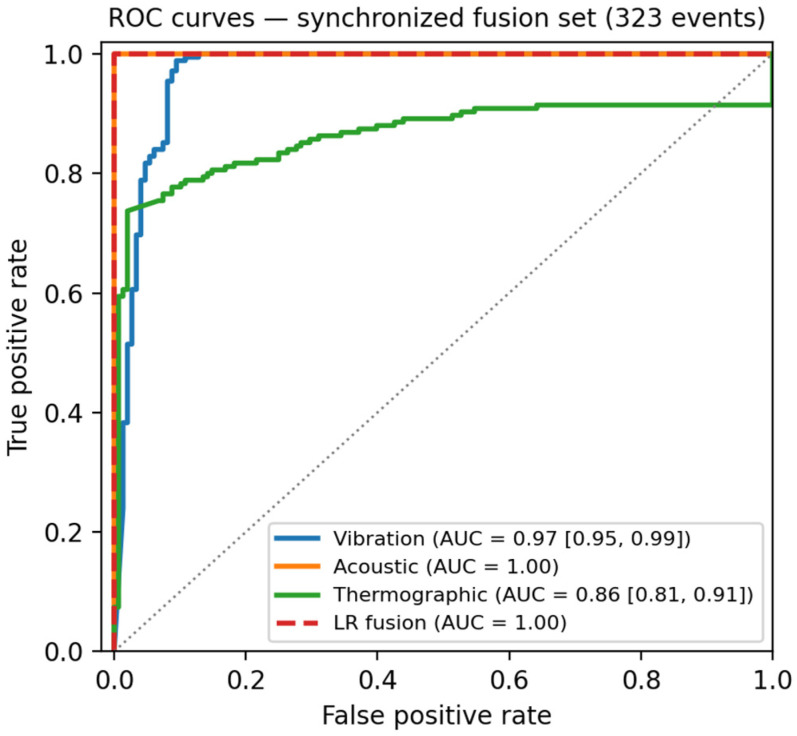
Per-modality ROC curves for the time-synchronized fusion set (323 co-occurring events), with AUC and 95% confidence intervals from a class-stratified bootstrap. Acoustic and logistic-regression fusion reach AUC = 1.00; vibration, after per-installation baseline recalibration, reaches AUC = 0.97 [0.95, 0.99], while thermography reaches 0.86 [0.81, 0.91].

**Figure 13:**
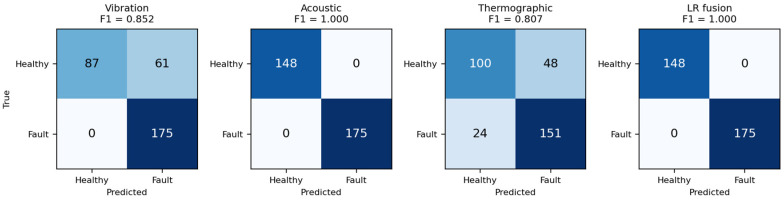
Confusion matrices on the time-synchronized set (148 healthy, 175 faulty events) for each single modality and the logistic-regression fusion. Vibration yields 61 false positives with no missed fault (F1 = 0.852); thermography yields 48 false positives and 24 missed faults (F1 = 0.807); and the fusion recovers all of these for a perfect confusion matrix (F1 = 1.000).

Regarding these synchronized events, the vibration autoencoder—trained on the first session, with only its input scaler and 95th-percentile threshold re-estimated from the 44 in-regime healthy windows recorded before the first synchronized event, and no weight retraining (Section 6.4)—reaches F1 = 0.852 [0.827, 0.875] with perfect recall, and an acoustic classifier of F1 = 1.000; however, thermography alone only reaches F1 = 0.807 (AUC = 0.86 [0.81, 0.91]) with 48 false positives and 24 missed faults, as the balanced motor warms to 36–40 °C during prolonged operation, overlapping the fault range, and early fault windows are still thermally cold. The acoustic F1 of 1.000 here exceeds the 0.962 obtained in Section 5.4’s bootstrap; the two figures are not directly comparable, as Section 5.4 draws offline log-Mel scores across a severity-mixed bootstrap that injects false-positive variability, whereas the present figure is the in-pipeline classifier on this cleanly separated acquisition, and the jump to a perfect score should be read as further evidence of intra-session separability (Section 6.4) rather than of field generalization. The rule-based strategies inherit the thermographic weakness (logical OR F1 = 0.805; logical AND F1 = 0.926; majority vote F1 = 0.936). The logistic-regression meta-learner reaches F1 = 1.000 [1.000, 1.000] with all-positive per-modality weights (w_vib = +1.6, w_aco = +5.6, w_thm = +1.2), recovering exactly the 24 faults missed by thermography and the 61 false alarms produced by vibration. If the vibration threshold is instead frozen from the first session with no baseline recalibration, the channel degrades to F1 = 0.703 with each healthy event flagged; the meta-learner then withdraws its weight (w_vib = +0.10) and the fusion still reaches F1 = 1.000 on the strength of the remaining channels—an explicit measured instance of the redundancy argument developed below. Random Forest and gradient boosting match the logistic regression, whereas a shallow multilayer perceptron fails to converge on the small feature space, corroborating the low-capacity meta-learner adopted in Section 5.4 (Table 8). Per-modality ROC curves with bootstrap AUC confidence intervals (Figure 12) and the complete confusion matrices (Figure 13) for this synchronized set are reported alongside those of Section 5.4.

This synchronized analysis should be read as a consistency check rather than as primary evidence of complementarity. Its value is three-fold: it confirms that, on genuinely co-occurring observations, every modality receives a positive weight in the meta-learner and none acts as dead weight; it verifies that the learned fusion does not degrade relative to the single modalities; and it exercises the synchronization scheme that Section 5.4 identified as missing. It does not, on this acquisition, show the fusion exceeding the best single modality, because the acoustic channel alone already saturates at F1 = 1.000 on this cleanly separated data; the additive benefit of fusion over the best single baseline is instead the one quantified in Section 5.4 (mean F1 = 0.989 versus 0.962 for acoustic). The two experiments are therefore complementary rather than contradictory: Section 5.4 estimates the additive benefit in the regime where no single modality saturates; whereas the present synchronized set, on which the acoustic channel is already perfect, can only test—and does confirm—that the learned fusion preserves this performance without degradation. Accordingly, the main argument for retaining three modalities in deployment is robustness and redundancy rather than an additive score on this controlled set; acoustic dominance here is contingent on a quiet laboratory, and in the field, modality can be degraded (a microphone masked by ambient plant noise, or thermography confounded by warm-up transients), in which case the remaining channels preserve detection. A field demonstration of a case in which thermography or vibration uniquely captures a fault that the other channels miss, together with a cross-session, event-synchronized study on additional machines, is identified as the definitive validation and remains future work.

## 6. Discussion

### 6.1. Architectural Implications

The combination of vibration, acoustic, and thermographic sensing produced complementary evidence of failure: vibration captures the mechanical signature, acoustic captures incipient acoustic patterns that may emerge before vibration-detectable damage, and thermography captures the steady-state thermal footprint, which is robust to ambient mechanical noise. The late-fusion experiment of Section 5.4 provides preliminary evidence of this complementarity (mean F1 = 0.989 above every single-modality baseline in the non-saturated bootstrap; the event-synchronized analysis of Section 5.5 shows that this additive margin closes once the acoustic modality alone saturates, so in deployment the complementarity is valuable chiefly as robustness and redundancy), specific to the induced static-unbalance scenario evaluated here, in which all three modalities observe the same fault mode, and consistent in direction with results reported in [4] for vibration–acoustic data fusion in electric motors. Pushing inference to the edge implies that only high-level results are transmitted (anomaly flags, class labels, lightweight statistics), preserving bandwidth and privacy, which is particularly relevant in industrial environments with limited infrastructure or high data sensitivity. Beyond bandwidth and privacy, keeping inference and fusion at the edge is what makes the latency budget of a fault-to-actuation safety loop meaningful; once a fault is detected, an automatic machine stop can be triggered locally, within the millisecond-scale regime targeted by 6G URLLC, before secondary mechanical damage develops. This control loop does not traverse the cloud uplink, which the latency analysis of Section 6.3 shows to dominate the end-to-end budget; the practical relevance of URLLC-class latency therefore lies in this local edge loop rather than in the cloud path. Consistent with this scope, the present study is a proof-of-concept that demonstrates feasibility on a controlled single-machine testbed rather than an exhaustive, fully generalizable validation across industrial scenarios.

### 6.2. Adaptation to 6G: A Forward-Looking Roadmap

This subsection is a forward-looking design roadmap rather than a report of validated results: it describes how the present prototype is intended to migrate onto a 6G substrate and should be read as such. The only empirically validated 6G-relevant result of this work is the end-to-end URLLC latency experiment of Section 6.3, which quantifies why edge inference is a precondition for the URLLC capabilities of 6G to be observable at the application level. Although the prototype uses Wi-Fi and a local MQTT broker, the architecture is designed to evolve toward a 6G substrate incrementally, without rewriting the application plane. The six IMT-2030 usage scenarios [13] relate to the architecture chiefly through the hyper-reliable low-latency (HRLLC) scenario that underpins the edge safety loop discussed above; a full scenario-by-scenario mapping is beyond the scope of this proof-of-concept study and is left to future work.

### 6.3. End-to-End URLLC Latency Measurement

**Figure 14:**
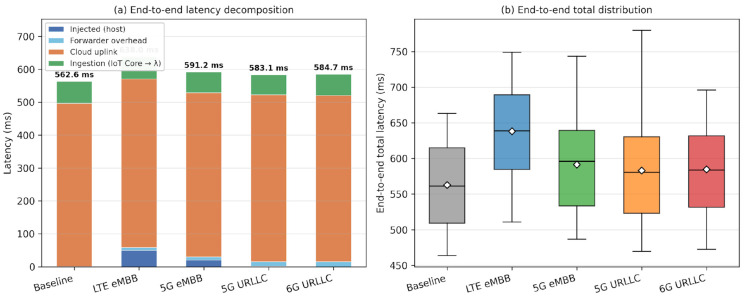
End-to-end latency decomposition of the forwarder → AWS IoT Core → Lambda pipeline under five injection profiles (*N* = 50 events per profile, 10 Hz, ≈258-byte payload, route Tucumán → AWS US-East-2). (**a**) Stacked-bar decomposition per leg: synthetic injection on the host, forwarder overhead, cloud uplink (forwarder → IoT Core via TLS) and ingestion (IoT Core rule → Lambda dispatch). (**b**) Full distribution of total end-to-end latency. The cloud-uplink leg (~500 ms) dominates the budget across all profiles, neutralizing the differences between LTE, eMBB and URLLC radio-access regimes.

To complement the architectural discussion with a quantitative measurement, a controlled latency-injection experiment was carried out on the deployed prototype. A Python forwarder receives every event published to the local Mosquitto broker, applies a per-event Gaussian delay sampled from *N*(μ, σ^2^) with μ = mean delay and σ = jitter, and republishes the message to AWS IoT Core over TLS-MQTT. An AWS IoT Core rule enriches each message with the server-side ingestion timestamp and forwards it to an AWS Lambda function, which timestamps the arrival and persists each event to a dedicated DynamoDB table. Five injection profiles are evaluated, each over 50 events at 10 Hz with an approximately 258-byte JSON payload: baseline (no injection), legacy LTE eMBB (μ = 50 ms, σ = 10 ms), 5G eMBB (μ = 20 ms, σ = 4 ms), 5G URLLC (μ = 1 ms, σ = 0.2 ms), and 6G URLLC (μ = 0.1 ms, σ = 0.05 ms). The URLLC profiles correspond to user-plane latency targets specified by 3GPP TS 22.261 [45] and Report ITU-R M.2410 [66]; the 6G targets are based on recent literature projections [14,67,68]. The methodology corresponds to a software shim that isolates the effect of one-way transport latency on the end-to-end pipeline while keeping the application plane unchanged across runs. The deployment site is Tucumán, Argentina, and the AWS region is US-East-2 (Ohio), separated by approximately 9000 km. Table 9 reports the measured end-to-end latency, and Figure 14 decomposes it per pipeline leg.

Three observations follow: First, the injection mechanism is faithful for eMBB-class profiles (<1% error on the mean, <6% on the standard deviation). Second, the host scheduler saturates the sub-millisecond regime: for the 5G and 6G URLLC profiles, the forwarder overhead (~15 ms) saturates regardless of the configured target, attributable to the Windows scheduler timer resolution (~15.625 ms per tick). Third, and most relevant, the cloud-uplink leg dominates the latency budget: the forwarder → AWS IoT Core segment contributes consistently between 495 and 513 ms across all profiles, between 79% and 88% of the total. This is the combined effect of the ~9000 km geodesic distance, mutually authenticated TLS session establishment, and propagation through intermediate networks not optimized for industrial traffic. Total end-to-end latencies converge to the 563–638 ms interval, and the difference between the means of 6G URLLC (584.67 ms) and baseline (562.63 ms) is statistically indistinguishable from the cloud-uplink noise. A hypothetical three-order-of-magnitude improvement in radio-access latency (from 50 ms to 0.1 ms) translates to an end-to-end improvement below 9% when processing occurs in a distant cloud region. This has a direct architectural implication: even with a 0.1 ms URLLC access link, the only viable route to meet sub-100 ms end-to-end budgets at industrial scale is to run inference locally on the device. The TinyML paradigm adopted here is therefore not an optional optimization but a necessary condition for the URLLC capabilities promised by 6G to manifest as observable application-level improvements. The injection mechanism is faithful only for eMBB-class profiles; for the 5G and 6G URLLC profiles, the host timer resolution (~15.6 ms tick) saturates the configured sub-millisecond targets, so these profiles are not independently resolved. The URLLC conclusion therefore follows from the magnitude of the cloud-uplink leg (~500 ms) rather than from sub-millisecond injection fidelity, and holds a fortiori for any radio-access regime. The US-East-2 region was used for deployment; because the conclusion is dominated by the cloud-uplink leg, it would also hold for a geographically nearer region such as sa-east-1.

### 6.4. Limitations

The current evaluation is restricted to a single motor under controlled conditions with a relatively limited dataset; generalization to other rotating machines will require additional data or transfer-learning strategies. Two modalities reach near-perfect scores on the present validation sets: the thermographic CNN reaches values close to 100% on accuracy and the acoustic classifier achieves F1, precision, recall, and AUC all equal to 1.00. These results reflect the strong intra-modality separability of the present dataset more than fully established generalization. A more fundamental concern affects both modalities: the acoustic dataset was captured in a single session (the motor was first run balanced, then the disk was unbalanced and the recording continued; the Edge Impulse 65%/35% split was obtained by cutting each of these long recordings into a training and a test segment) and the thermographic dataset has an analogous structure (one video balanced, one video unbalanced, both on the same day, frame-level train/test split). In both modalities, training and test partitions therefore share the same recording session, ambient environment, sensor placement and motor warm-up state, with adjacent frames highly correlated. The F1 = 1.00 and the near-100% CNN accuracy demonstrates separability of the present recordings but do not establish session-to-session generalization. A cross-session validation protocol with independently captured sessions will be incorporated in future work. In this respect, the measurements analyzed in Section 5.5 were captured in a separate session, on a different day but on the same bench, from the benchmarks of Section 5.1, Section 5.2 and Section 5.3. However, the two sessions differ in acoustic and thermographic instrumentation (microphone sampling rate, and thermal-camera resolution and rendering), so they do not constitute a controlled cross-session pair; a leave-one-session-out and leave-one-machine-out evaluation with matched instrumentation therefore remains necessary future work. We further note that the vibration detector is a one-class model whose healthy baseline is, by construction, calibrated on the target machine: per-machine healthy calibration is intrinsic to its deployment and is precisely what the within-session evaluation reflects, rather than an unconstrained transfer of a model across machines without recalibration. For the vibration modality, whose instrumentation is identical in both sessions (the same Nicla Sense ME remounted on the same bench), the two acquisitions do constitute a controlled cross-session pair, and the leave-one-session-out evaluation was carried out. Transferring the frozen session-1 model to session 2 preserves fault recall (100% of unbalance windows flagged) but collapses on the healthy class: 99% of healthy windows exceed the frozen threshold. The shift is attributable to the remount rather than to machine condition: the dimensionless shape descriptors (crest, impulse and shape factors) transfer within ±2%, whereas the location and asymmetry statistics shift substantially (mean −10%, median −18%, and a sign change in skewness), the signature of a changed gravity projection on the sensor axes, compounded by a 16–23 °C ambient ramp during the second recording. Re-estimating only the input scaler and the 95th-percentile threshold on a healthy baseline of the new installation—the per-machine calibration that, as noted above, is intrinsic to one-class deployment and standard practice in condition monitoring—restores F1 = 0.9751 [0.961, 0.987] with perfect recall at the window level using a 12 min baseline, and F1 = 0.852 at the event level of Section 5.5, where only 44 in-regime healthy windows preceded the synchronized campaign. Model weights are never retrained. The corresponding leave-one-session-out evaluation for the acoustic and thermographic modalities, with matched instrumentation, remains future work. The vibration benchmark of Section 5.1 is less exposed because the ten statistical features are intrinsically frequency-independent, but it is also derived from a single test bench and the cross-machine caveat applies. An additional physical constraint affected the severe-unbalance case MA: the thermographic recording could only be sustained for 43 s because operating the bench at MA for longer intervals risked structural damage. This is not a methodological choice but a physical limitation that strengthens the motivation for the proposed PdM architecture: the cases in which intervention is most urgent are precisely those for which extended data collection is unsafe, favoring detectors calibrated on the more abundant normal and moderate-unbalance regimes. More generally, the near-perfect single-session scores should be read as evidence of intra-session separability, carrying a correspondingly high risk of overfitting and information leakage, rather than as validated performance; the appropriate next step is an explicit leave-one-session-out and leave-one-machine-out evaluation on independently captured sessions and additional machines, which the present single-session, single-machine data do not permit. The thermographic acquisition and network configuration were selected to fit the embedded target and follow standard lightweight-CNN practice rather than being obtained through a systematic acquisition or hyperparameter search; a principled ablation, together with thermography-specific robustness-oriented architectures such as attention-based networks [65], is left for future work. The benchmark is moreover limited to static rotor unbalance: extending the system to other characteristic faults (bearing defects, shaft misalignment, mechanical looseness and gear defects) will require datasets containing those failure modes and, for bearing-defect signatures in particular, a higher effective vibration sampling rate than the present host-side acquisition cadence allows, which currently places the Nyquist limit below the relevant defect frequencies. Finally, the present study reports per-inference energy and memory figures but not a system-level energy budget; a complete per-node power characterization including sensing, radio and duty cycle is identified as future work, consistent with the energy-efficiency goals of TinyML and Industry 5.0.

### 6.5. Preliminary Per-Node Energy Budget

Building on the energy-efficiency considerations noted above, this subsection provides a preliminary per-node power estimate derived from component datasheets (Table 10), assuming a common 3.3 V rail; full instrumented characterization remains for future work. Two observations follow: First, the two nodes built around 480 MHz Arm Cortex-M7 processors, namely the thermographic node (OpenMV Cam H7+) and the Portenta H7 gateway, dominate the budget, each drawing on the order of 0.4–0.6 W when active, with the FLIR Lepton adding ~150 mW of continuous sensing power and periodic flat-field-correction peaks of ~650 mW. For the thermographic node, this host-processor cost is dominated by the classifier itself: a single 96 × 96 × 3 floating-point inference takes ~462 ms on the H7+ (Table 6), so the Cortex-M7 is occupied almost continuously at the ~2 frames/s achievable on-device. Second, and consistent with the latency analysis of Section 6.3, the energy is dominated by the host processors and the Wi-Fi radio, whose transmit bursts reach ~370 mA, rather than by inference on the dedicated low-power accelerators: the in-sensor acoustic decision costs only 5.55 µJ and the vibration autoencoder occupies the Cortex-M4F for just 254 µs per inference. The principal energy levers are therefore lower-power host processors, aggressive duty-cycling of the camera and radio, and minimizing cloud transmission, and not a reduction in the inference cost on the dedicated accelerators, which is already negligible.

## 7. Conclusions and Future Work

This article presented and experimentally evaluated a novel multi-sensor edge architecture for industrial predictive maintenance based on TinyML, with an explicit forward-looking discussion of its evolution path toward 6G. Three sensing modalities (vibration, acoustic, and thermography) were deployed on commercial off-the-shelf hardware connected through an I^2^C-based serial link to an Arduino Portenta H7 gateway and a serverless AWS IoT back-end. On the vibration modality, five anomaly-detection algorithm variants were trained and compared on real signals from a controlled unbalance test bench; four of the five reached an F1-score above 0.98, PCA reached only F1 = 0.669 because a linear one-class detector reconstructs the moderate-unbalance regime almost as well as normal operation, and the INT8-quantized FC autoencoder (Q8INT) emerged as the recommended deployment default with F1 = 0.9807, with 254 µs of inference latency and 6056 B of Flash on the target Cortex-M4F. A controlled leave-one-session-out transfer of this detector to a second acquisition with the remounted sensor preserves perfect fault recall and, after a short per-installation healthy-baseline recalibration with frozen weights, restores F1 = 0.975. The acoustic modality was addressed with a classifier on log-Mel filterbank energies (the native feature representation of the Syntiant NDP120 in-sensor neural decision coprocessor) and the thermographic modality with a lightweight binary CNN. A preliminary intra-session late-fusion experiment provided initial evidence of inter-modality complementarity, where a logistic-regression meta-learner on the three confidence scores reached mean F1 = 0.989 [0.980, 1.000] on a class-stratified bootstrap, which was higher than every single-modality baseline and every rule-based fusion strategy in the non-saturated regime; conversely, on an event-synchronized acquisition (Section 5.5), the acoustic channel alone already reached F1 = 1.000, so the principal demonstrated benefit of the multimodal design was robustness and redundancy rather than an additive gain, with cross-session confirmation left to future work. A controlled URLLC latency-injection experiment showed that the cloud-uplink leg dominates the end-to-end budget by 79–88%, demonstrating that edge inference is a necessary condition for the URLLC capabilities promised by 6G to manifest at the application level. All connectivity in the present prototype is realized over Wi-Fi and MQTT; no 5G or 6G modems or radios were used, and no real-radio testing was performed, so 6G compatibility is presented as a design trajectory (Section 6.2) rather than a tested capability. The findings should be interpreted within the limitations of Section 6.4 (single-motor, intra-session leakage in the acoustic and thermographic datasets), and the following several research directions emerge: split federated learning across nodes, gateways, and clouds by leveraging communication-efficient variants, such as PipeSFL and CSE-FSL, for heterogeneous clients [74,75]; neuromorphic acceleration of sparse, event-driven inference on coprocessors, such as BrainChip AKD1000 or Innatera Pulsar, integrated with a Jetson-class host; post-quantum cryptography on gateways and selected nodes (e.g., ML-KEM-768) [76,77,78] to secure 6G era industrial IoT links against “harvest-now, decrypt-later” attacks; and selective state-space models (e.g., Mamba) for long vibration and acoustic sequences; and ambient-IoT operation of the sensing tier, building on the battery-less device classes under 3GPP study [79,80].

## Figures and Tables

**Table 1:** Hardware specification used in each sensing modality, embedded processing, and IoT gateway.

Function	Component	Model/Reference	Key Features
Vibration	Smart node with triaxial MEMS accelerometer	Arduino Nicla Sense ME (Bosch BHI260AP)	Host MCU: nRF52832 (ARM Cortex-M4F at 64 MHz, FPv4-SP); runs TinyML vibration models locally
Acoustic	Acoustic node with neural decision coprocessor	Arduino Nicla Voice (Syntiant NDP120 ASIC)	Acoustic DSP + Syntiant Core 2 neural engine; in-sensor log-Mel classification
Thermography	Embedded thermal camera + IR microbolometer	OpenMV Cam H7+ with FLIR Lepton	Binary CNN inference on radiometric images using FP32
IoT gateway	Embedded gateway with wireless connectivity	Arduino Portenta H7	Dual-core MCU (Cortex-M7/M4); Eslov–Wi-Fi/MQTT bridge to AWS IoT
Inter-device link	I^2^C-based serial connector	Eslov (Arduino Pro)	Up to 3.4 Mbit/s in I^2^C High-Speed mode; far above accelerometer throughput
Cloud platform	Serverless IoT, storage, and inference services	AWS (IoT Core, Lambda, DynamoDB, S3, SageMaker, TwinMaker, Grafana, SNS)	Storage, alerting, digital twin, and centralized training

**Table 2:** Summary of the measurement dataset: sensing nodes, signal representations, recording durations (mm:ss), and partitioning for the three operating conditions (normal (N); moderate unbalance, abnormal (A); severe unbalance, severely abnormal (MA)). The bottom row summarizes the event-synchronized fusion set of Section 5.5. The vibration row lists both acquisition sessions: session 1 (S1) feeds Section 5.1’s benchmark and model training; and session 2 (S2), recorded on a different day with the remounted sensor, feeds the synchronized fusion set and the cross-session evaluation (Section 5.5 and Section 6.4). Acoustic and thermographic rows correspond to the single continuous recordings analyzed in Section 5.2 and Section 5.3.

Modality	Sensor Node and Signal	Window/Features	Duration N/A/MA	Windows or Events N/A/MA	Partition
Vibration	Nicla Sense ME (BHI260AP), triaxial accel., 10 Hz host cadence	6 s window (60 samples); 10 time-domain features	S1: 13:04/12:49/7:55; S2: 24:25/22:43/0:54	S1: 129/126/78; S2: 235/227/8 windows of 6 s	Chronological 60/20/20 on S1 healthy (one-class); S2 held out (Section 5.5 and Section 6.4)
Acoustic	Nicla Voice (NDP120), MEMS mic, 16 kHz mono, 16-bit	40 × 40 log-Mel map (~0.96 s), 40 Mel bins	18:01/19:16/1:07	1126/1204/69 events	5-fold stratified CV
Thermography	OpenMV H7+ with FLIR Lepton 3.5, 160 × 120, ~30 fps	96 × 96 frame; whole-frame temperature (1 fps sampled)	23:50/23:02/0:59	1430/1055/59 frames	5-fold CV, frame-level
Synchronized fusion set (Section 5.5)	All three, wall-clock aligned	6 s co-occurring events	Overlapping operating interval	323 events (148 healthy/175 fault)	Class-stratified bootstrap + OOF CV

**Table 3:** Ten time-domain statistical features extracted from each 6 s vibration window (*N* = 60 samples at the 10 Hz host cadence). Features 1–5 are amplitude/energy descriptors; features 6–10 are dimensionless shape ratios invariant to amplitude scaling.

Statistical Feature	Associated Equation
Mean	$\mu =\frac{1}{N}\sum x_{i}$
Standard deviation	$\sigma =\sqrt{\frac{1}{N-1}\sum {\left(x_{i}-\mu \right)}^{2}}$
Root mean square	$x_{RMS}=\sqrt{\frac{1}{N}\sum x_{i}^{2}}$
Peak amplitude	$x_{pk}=\underset{i}{max}\left|x_{i}\right|$
Peak-to-peak	$x_{pp}=\underset{i}{max}x_{i}-\underset{i}{min}x_{i}$
Skewness	$\gamma_{1}=\frac{1}{N\sigma^{3}}\sum {\left(x_{i}-\mu \right)}^{3}$
Excess kurtosis	$\gamma_{2}=\frac{1}{N\sigma^{4}}\sum {\left(x_{i}-\mu \right)}^{4}-3$
Crest factor	$CF=\frac{x_{pk}}{x_{RMS}}$
Shape factor	$SF=\frac{x_{RMS}}{\frac{1}{N}\sum \left|x_{i}\right|}$
Impulse factor	$IF=\frac{x_{pk}}{\frac{1}{N}\sum \left|x_{i}\right|}$

**Table 4:** Performance comparison of the five anomaly-detection algorithm variants on vibration data, with per-sample inference times on the reference laptop and the Cortex-M4F of the Nicla Sense ME, and Flash and stack footprints from the Arduino IDE build report and from -fstack-usage. The two FC autoencoder rows correspond to the same trained network exported in FP32 and INT8 (Q8INT) form. F1 and accuracy are computed on strictly non-overlapping 60-sample windows under a chronological 60/20/20 healthy split (Section 5.1); latency and memory figures correspond to the deployed on-target artifacts. The Isolation Forest row reports the 100-estimator offline model; its MCU latency corresponds to the distilled 6-tree implementation (Section 4.2).

Model	F1	Accuracy	Laptop/Sample (µs)	MCU/Sample (µs)	MCU Flash (B)	MCU Stack (B)
FC autoencoder Q8INT (TFLite Micro)	0.9807	0.9651	1.28	254	6056	1132
FC autoencoder FP32 (TFLite Micro)	0.9807	0.9651	2.40	293	6640	1224
One-Class SVM	0.9831	0.9694	32.37	7057	14,584	40
Isolation Forest	0.9878	0.9782	0.31	32	14,308	28
PCA	0.6689	0.5590	0.18	13	132	64

**Table 5:** Configuration of the Edge Impulse “log-bin (NDP120/200)” feature extractor, classifier architecture, and validation-set performance of the int8-quantized acoustic classifier deployed on the Syntiant Core 2.

Parameter	Value
Frame length/stride	32 ms/24 ms (8 ms overlap)
Mel filterbank size/FFT length	40 filters/512 samples
Pre-emphasis coefficient	0.96875
Output representation	40 log-Mel bin energies per frame (no DCT)
Classifier architecture	40 × 40 × 1 input; 2 × Conv2D (8 filters, 3 × 3, ReLU) + 2 × 2 max-pool; dropout 0.25; flatten; dense softmax (2 classes)
Training epochs/optimizer/learning rate	20/Adam/0.0005
Loss function/data augmentation	Categorical cross-entropy/none
F1/accuracy/precision/recall/AUC (int8)	1.00/1.00/1.00/1.00/1.00
False positives/negatives	0%/0%
Inference cadence on Syntiant Core 2	24 ms per decision (~42 inferences/s)
Model parameter memory (NDP120_B0)	1.375 KB of 640 KB (~0.2%)
Estimated energy per inference (0.9 V)	5.55 µJ
Total acoustic collected (train/test split)	19 min 48 s (65%/35% by duration)

**Table 6:** Architecture and training configuration of the binary thermographic CNN classifier deployed on the OpenMV Cam H7+.

Parameter	Value
Input	96 × 96 × 3 RGB, normalized to [0, 1]
Architecture	3 × Conv2D (16/32/32 filters, 3 × 3, ReLU) + 2 × 2 max-pool; flatten; dense 64 (ReLU); dense 1 (sigmoid)
Output	Single sigmoid fault probability (decision threshold 0.5)
Parameters/precision	~219 k/32-bit floating-point
Optimizer/loss	Adam/binary cross-entropy
Training epochs/regularization	50/dropout (rates 0.5 and 0.3) and early stopping (best weights restored)
Train/test split	Frame-level over two single-session videos
Total frames collected (train/test)	12,455 frames (80%/20%, stratified)
F1/accuracy/precision/recall/AUC	1.00/1.00/1.00/1.00/1.00
False positives/negatives	0%/0%
Inference time on OpenMV Cam H7+	~462 ms per frame (~2.2 frames/s)

**Table 7:** Late-fusion performance of the three modalities under a 1000-iteration class-stratified bootstrap (150 events per iteration: 50 normal, 100 fault). Means are reported; the F1 column includes 95% confidence intervals from the bootstrap distribution; the confusion column reports mean TN/FP/FN/TP per iteration. The vibration baseline is the FC autoencoder of Section 5.1, retrained under the corrected non-overlapping windowing protocol.

Model	Accuracy	Precision	Recall	F1 [95% CI]	TN/FP/FN/TP
FC autoencoder (vibration)	0.797	0.892	0.793	0.839 [0.788, 0.885]	40/10/21/79
Acoustic only (log-Mel + LR)	0.947	0.927	1.000	0.962 [0.939, 0.985]	42/8/0/100
Thermographic only (textures + LR)	0.765	0.740	1.000	0.850 [0.830, 0.873]	15/35/0/100
Fusion—logical OR	0.732	0.714	1.000	0.833 [0.816, 0.855]	10/40/0/100
Fusion—logical AND	0.855	0.987	0.793	0.879 [0.830, 0.920]	49/1/21/79
Fusion—majority vote (≥2 of 3)	0.922	0.895	1.000	0.945 [0.917, 0.971]	38/12/0/100
Fusion—logistic regression (5-fold CV)	0.985	0.979	1.000	0.989 [0.980, 1.000]	48/2/0/100

**Table 8:** Comparison of late-fusion meta-learners on the time-synchronized set of 323 events (Section 5.5), with point estimates from out-of-fold stratified five-fold cross-validation. Because the acoustic channel already separates the classes on this acquisition, all viable combiners reach the 1.000 ceiling, so the comparison only shows that no higher-capacity model is required and provides no ranking among them. The shallow MLP collapses to the majority-class (all-negative) prediction; this is a training-optimization failure (degeneration) on a small, class-imbalanced three-feature set and is not overfitting. The approximately four-parameter logistic regression is retained on capacity, stability, and interpretability grounds.

Late-Fusion Meta-Learner	Accuracy	Precision	Recall	F1
Logistic regression (adopted)	1.000	1.000	1.000	1.000
Random Forest	1.000	1.000	1.000	1.000
Gradient boosting (XGBoost/HGB)	1.000	1.000	1.000	1.000
Shallow MLP (16-8)	0.458	0.000	0.000	0.000

**Table 9:** End-to-end latency from forwarder publish to AWS Lambda arrival under five injection profiles (50 events per profile, *N* = 250). All 250 events were matched at 100% between local forwarder log and DynamoDB ingestion. Latencies are in milliseconds.

Injection Profile	*n*	Mean	Std	p50	p95	p99
Baseline (no injection)	50	562.63	59.35	561.24	649.59	662.78
Legacy LTE eMBB (μ = 50, σ = 10)	50	637.97	63.61	638.61	732.41	744.23
5G eMBB (μ = 20, σ = 4)	50	591.23	65.42	596.07	686.00	720.84
5G URLLC (μ = 1, σ = 0.2)	50	583.09	69.46	580.75	669.78	762.13
6G URLLC (μ = 0.1, σ = 0.05)	50	584.67	61.45	584.07	673.28	695.26

**Table 10:** Preliminary per-node power estimate derived from component datasheets, assuming a common 3.3 V supply. Values are datasheet-based estimates pending instrumented measurement.

Node	Dominant Component(s)	Datasheet Figure (Source)	Estimated Active Power
Vibration node (Nicla Sense ME)	nRF52832 host + BHI260AP	3.7 mA run @ 64 MHz [69]; 0.25–0.39 mA AI @ 25–50 Hz [70]	~14 mW
Acoustic node (Nicla Voice)	Syntiant NDP120 + nRF52832 host	5.55 µJ per inference (~0.2 mW avg); host 3.7 mA [69]	~13 mW
Thermographic node (OpenMV Cam H7+)	STM32H743 @ 480 MHz + FLIR Lepton 3.5	~132 mA (275 µA/MHz) [71]; Lepton ~150 mW, ~650 mW FFC [72]	~0.45–0.59 W
Gateway (Portenta H7)	STM32H747 @ 480 MHz + CYW4343W Wi-Fi	M7 ~132 mA [71]; Wi-Fi up to ~370 mA TX [73]	~0.43 W + bursty TX

## Data Availability

Datasets generated and analyzed during the study are available from the corresponding author on reasonable request.
